# How group coaching contributes to organisational understanding among newly graduated doctors

**DOI:** 10.1186/s12909-020-02102-8

**Published:** 2020-06-16

**Authors:** Bente Malling, Lydia de Lasson, Eva Just, Nikolaj Stegeager

**Affiliations:** 1 Centre for Health Sciences Education, Faculty of Health, Aarhus University, Palle Juul-Jensens Boulevard 82, 8200 Aarhus N, Denmark; 2 Department of Anaesthesiology and Intensive Care, Aarhus University Hospital, Palle Juul-Jensens Boulevard 99, 8200 Aarhus N, Denmark; 3 Consulting Company Justeva, Aldersovej 23 D, Aarhus N, Denmark; 4 Department of Learning and Philosophy, Aalborg University, Kroghstræde 3, 9220 Aalborg, Denmark

**Keywords:** Specialist training, Professional development, Leadership development, Group coaching, Qualitative study

## Abstract

**Background:**

Practising medicine at an expert level requires skills beyond medical expert knowledge. Research shows that newly appointed consultants feel less prepared to deal with leadership issues compared to issues regarding medical expertice. Newly graduated (NG) doctors and residents in particular struggle with leadership and organisational issues.

The purpose of this study was to explore the impact of group coaching on NG doctors’ approach to organisational and leadership challenges in daily practice during the transition period from medical school to clinical work.

**Methods:**

Newly graduated doctors participated in a group coaching course comprising three full-day sessions and five two-hour sessions. The purpose was to support NG doctors’ professional development regarding organisational issues in the first years after graduation. The coaches were health professionals with certified coaching training. Data from the intervention were collected from open-ended questionnaires and observational notes. A thematic analysis was performed.

**Results:**

Forty-five NG doctors participated in a total of six courses sharing experiences and problems reflected in their professional lives. The following themes emerged: Revelation of the hidden curriculum, importance of professional relations, inter-professional communication, conflict management and emerging leadership skills. Participants’ communication skills improved due to an increased awareness of other peoples’ perspectives and preferences. They realized the importance of good relations, saw how they could become active contributors in their departments and began to practice leadership skills through e.g. involvement of the team, delegation of work and negotiation of own obligations.

**Conclusion:**

Newly graduated doctors seemed to develop a growing awareness of organisational issues and a deeper understanding of the complexity of health care organisations through participation in a group coaching course. The study indicates that participation in a group coaching course probably contributes to improve practice among NG doctors. Further studies are needed to consolidate the findings and explore possible organisational effects.

## Background

Internationally, blueprints in specialist training of doctors stress the importance of gaining medical expert competence, as well as acquiring a broad range of competencies such as collaboration skills, intercollegial and interprofessional communication and leadership skills coupled with organisational knowledge and systems thinking, [1–4]. To practice at an expert level, doctors need to develop understanding of the organisation, communication and leadership skills [5–11]. Therefore, teaching leadership and understanding of the organisation should be part of specialist training. However, studies show that newly appointed consultants feel less prepared to deal with leadership issues as well as managing budgets and resources compared to their medical obligations [7, 8, 12]. Thus, there seems to be a need to find supplemental ways of teaching leadership and to obtain understanding of the organisation during specialist training.

The literature on the transition from being a medical student to working as a doctor primarily focus on managing responsibility and uncertainty, dealing with death or seriously ill patients, and struggling to develop a professional identity [13–18]. Many newly graduated (NG) doctors expect themselves to be able to take the lead in patient care [18]. However, taking the lead in patient care requires skills such as time management, professional communication skills and administrative skills [6, 19–21]. The NG doctors have usually not had much opportunity to practice these skills before entering the workplace. Thus, several researchers have suggested that training in organisational matters should start at the beginning of their professional career [22, 23].

Previous studies show that group coaching supports the formation of NG doctors’ professional identity (thinking, feeling and acting as a doctor) as well as their adaptation to the medical culture and a healthy work/life integration [18, 24]. However, these studies do not describe if and how group coaching influences NG doctors’ ability to adapt to health care organisations and the specific culture of these organisations.

## Methods

The purpose of this study was to explore the impact of group coaching on NG doctors’ way of managing organisational challenges in daily practice during their transition into practice.

The research question was “Does group coaching influence newly graduated doctors’ organisational understanding and competence?”

### Context of the study

The study took place in Denmark where we have outcome-based education and have adapted the seven CanMEDS roles. Specialist training includes one foundation year (two employments of six months each at two different hospital departments or one at a hospital department and one in general practice) and one year in an introductory position before entering specialist training. All postgraduate trainees are appointed an educational supervisor who is responsible for the educational programme of the trainee. However, all doctors in a department are obliged to act as clinical supervisors in daily clinical practice.

### Study design

Qualitative study using observations (field notes from coaching sessions) and written evaluations from participants, midterm and end of course evaluations. The evaluation forms are provided in an additional file. [See Additional file 1].

### Sampling and recruitment

Participants (NG doctors in their first working year) were invited by mail to attend a group coaching course. Participants were enrolled on a first come first served basis and distributed over the six courses in the study period (two years). The participants were employed at different hospital departments or in general practice. All participants provided informed consent and were guaranteed that data would be anonymised before publication. Quotes from participants were given pseudonyms. The study was exempted from ethics approval according to the Act on Research Ethics Review of Health Research Projects [25]. The Central Denmark Region Committee on Health Research Ethics was notified (1–10–72-6-16). [See Additional file 2].

### Intervention

All participants attended a full group coaching course. Table 1 presents the duration and content of the course. The purpose of the course was to support participants’ professional development in the transition from medical student to doctor in practice. The coaches used a systemic coaching model [26]. A typical group coaching session consisted of a dialogue between the coach and the person being coached (the coachee) as well as input from the group (reflecting team). Each participant was obliged to take on the position as coachee at least once during the course. Two coaches, both health professionals with specialized coaching training, taught the course and were present during the entire course.

**Table 1:** Duration, content, form and evaluation of the group coaching course offered to newly graduated doctors.

Duration	Three full-day sessions and five two-hour sessions over a four-month period
Content	Systemic coaching model Conflict management Awareness of and respect towards the perspective of others
Format	Coaching sessions Brief presentations Self-study of literature (handed out) between sessions Practice in daily work (Ex conflict management, communication strategies like asking questions, staying curious etc)
Evaluation	Written evaluations at midterm and end of course (forms shown in Additional file 1)

### Data collection

In each session one of the two coaches observed and made field notes comprising conversational themes, important comments from participants, interventions made by the coach and own reflections. Midway through the course and immediately after the last session, participants filled in evaluation forms consisting of five and ten open questions, respectively. Answers on selected questions from these evaluations were included in the analysis. The specific questions used, are presented in Table 2. The evaluation forms are provided in an additional file [See Additional file 1].

**Table 2:** Questions selected from Midterm and End-of course evaluation used in the study.

Questions from the Midterm evaluation	
How does participation in this group coaching course influence your daily work life (give specific examples)?	
Questions from the End of course evaluation	
How did participation in the group coaching course influence your thoughts and actions in your interaction with patients and relatives?	
How did participation in the group coaching course influence your thoughts and actions in your interaction with colleagues in the department?	
How did participation in the group coaching course influence your thoughts and actions regarding your interaction with other collaborative partners?	
How will you describe the importance of being in a group regarding your benefit from participating in the course?	

### Data analysis

A generic thematic analysis was applied [27]. All authors used an inductive approach to search for themes and provide preliminary codes of both the questionnaire data and the field notes from the first four groups. The authors compared and discussed the themes and the coding until consensus was obtained regarding themes and codes. The analysis resulted in six themes. The themes “Professional identity formation”, “Career planning” and “Work/life integration” have been reported elsewhere [18]. The remaining three themes and categories relevant for the present study are shown in Table 3.

**Table 3:** Themes and categories from the analysis with illustrating examples

Themes	Categories	Examples
The hidden curriculum	How things work here	“You feel kind of stupid, when you do not know how things work here” [Louise]
	What is said and what is actually meant	“We do not have assigned seats” (but do not choose the black chair)” [Julie]
Inter-professional communication, conflicts and relations	What other staff do	“Actually, I do not know what other staff do” [Peter]
	How to ask for help and feedback	“I felt alone” [Jane]
	How to ask questions on others’ perspectives	“It was helpful to ask questions to get to know the other party’s perspective” [Susanne]
Emerging leadership	Delegation	“I do not know what I can delegate and to whom” [Lone]
	Prescription and asking other staff to comply with orders	“It was difficult to make prescriptions in a proper way” [Trine]
	Take the lead in patient care	“What can I decide – and what should I leave to others to decide?” [Hanne]
	How to influence own working conditions	I now carefully consider: “Are there too many tasks or are the tasks too difficult” [Laura]

## Results

Forty-five doctors participated in six group coaching courses (38 females, 7 males). Findings are reported below under the headings, “The hidden curriculum”, “Relations, inter-professional communication and conflicts”, “Leadership skills” and “Development of organisational understanding through the coaching intervention”.

### The hidden curriculum

*“Becoming a doctor is much like climbing a wall. It requires far more than just acquiring and applying academic knowledge. You have to relate to nurses, senior doctors and the entire organizational setup”[Laura].*

All departments have a formal introductory program and supervisor system to coordinate the interplay between a newly employed and the organization. However, embedded norms, rules and the culture of a department are implicit knowledge, and are seldom revealed in the introduction leaving the NG doctors with a feeling of inferiority.

*“You feel kind of stupid when you do not know how things work here”[Louise]*

Newcomers are normally introduced to the head of the department and the formal organisational structure. However, knowledge such as “Who is the extremely helpful senior doctor” or “Who is the less helpful senior doctor who has forgotten how it was to be young and inexperienced” are rarely provided during these formal introductions. Furthermore, the unrevealed dynamics between doctors and other staff has to be experienced for the NG doctor to be able to navigate in complex organisations such as a hospital department.

Thus, NGs struggle to bring their knowledge into play in the organizational context governed by a hidden curriculum barely presented to them. Moreover, many participants believe that they should be able to perform at specialist level from the beginning.

### Relations, inter-professional communication and conflicts

The NGs found it distressing to be a newcomer without established relational support. The social safety net of being a well-known and respected colleague is lost with every shift in employment. Conditions for establishing a new safety net during their short employments are completely different from the long-lasting social relations in medical school.

Inexperienced doctors depend on collaboration with nurses and other staff who are typically more experienced and familiar with underlying organisational values and routines.

*“Collaborators often have another approach to the patients as they have different tasks. This may give rise to contradictory interests and conflicts”[Peter]*

Some were reluctant to ask for advice from experienced nurses and preferred to try to hide their insecurity. They found it challenging to bring their medical knowledge into play, especially if their knowledge was different from the standard routines of the experienced nurses. In these incidents some expressed fear that nurses might not support them in the future due to this incident of conflicting views.

*“It may be dangerous to challenge the nurses”[Henrik]*

Another reason for their reluctance was the participants’ experience of isolation due to their solitary working conditions and their impression of what they named the nurses’ sense of community in practice.

Many participants expressed similar concern about approaching senior doctors on matters such as feedback on their performance, to voice perceived excessive amounts of clinical work or tough work schedules.

Furthermore, the participants found it hard to begin their postgraduate life with six-month employments. They would often arrive at a department with a high turnover and varying cultures (see above). They feared that missteps when trying to fit in, would stigmatize them from the onset of their short tenure.

*“As a newcomer in a department it is extremely important how you are perceived by your new colleagues and the leader. You quickly get a label, which is hard to change – and it is a catastrophe to get a bad label.” [Jane]*

### Leadership skills

The participants struggled to attain personal authority. They knew that they hold formal authority within the organisational hierarchy but not to which extent, and many found it difficult to take on leadership obligations among older and more experienced co-workers. Delegation of tasks was among the reoccurring themes regarding leadership. The NGs expressed insecurities and were afraid to produce conflicts and they felt that they lacked the proper communication skills to express and negotiate their decisions regarding patient treatment and subsequent delegation of tasks to other staff. They did not know what co-workers expected of them and found that expectations often seemed to depend on relations or the amount of work in a particular day. Many NGs were reluctant to ask about what was expected of them.

### Development of organisational understanding through the coaching intervention

In the coaching sessions, participants brought up current incidents from clinical practice. They explored and analysed the organizational complexity of the incidents in a structured way, thus realising the vast number of stakeholders, the importance of communication and collaboration between staff and of acknowledging the difference in stakeholder perspectives. Through the coaching sessions, participants learned from and helped each other to become active contributors and engage more fully with the departments with their current level of expertise.

*“Solutions to problems implemented in other departments have inspired problem-solving in my own department” [Gladys]*

Concurrently with their growing understanding of the governing principles behind complex organizations, they realized that it was possible to make changes in their working conditions, if they had the courage to take the lead. One participant described how she proposed and implemented a peer-mentoring program in addition to the formal supervisor system at the department.

In the coaching sessions, the participants in the reflecting team were trained to pay thorough attention to the dialogue between coach and coachee and simultaneously notice their own thoughts and feelings triggered by the dialogue. After the session, their observations were discussed with the other participants. This design aimed to create awareness of other peoples’ perspectives or preferences. The NGs realized that even though they all shared the same level of experience as each other, their perspectives on the themes varied considerably. As their professional identity and self-confidence grew, they felt that their communication skills improved. Thus, many reported to be more at ease asking for the opinion of an experienced nurse and they reported a gradual improvement in how they were able to bring their own experience and skills to contribute to patient care in a constructive and forward thinking manner.

*“Overall, I think that collaboration with doctors and nurses in the department have become easier - probably because of me staying curious and open towards the opinions of my co-workers” [Susanne]*

They stated that they felt the course helped them become more efficient employees. They learned which tasks to delegate and prioritize and they gradually found it easier to ask for help, support and feedback.

*“I have learned to analyse a distressing situation and sort out which kind of help is needed: Are there too many tasks or are the tasks too difficult?” [Laura]*

With this increase in self-confidence, participants gradually felt encouraged to exert leadership.

*“I am more confident being a doctor. I have improved my ability to delegate tasks, keep my promises and not being an excuse for myself” [Lisa]*

The awareness of the importance of recognising differences in perspective was helpful in managing conflicts. One participant told how she prevented the escalation of a potential conflict simply by asking the other party how they perceived the situation. The process of applying simple principles of conflict management, with discussions and shared reflections in the group afterwards, encouraged more participants to actively engage themselves in resolving potential conflicts. They learned that asking for other peoples’ perspective is not a sign of weakness but a powerful tool to prevent and dissolve potential conflicts.

*“Instead of a prefixed mind-set about other peoples’ thinking in a certain situation, I have become better at exploring their thoughts and at the same time I have made an effort to remember that my interpretation is not necessarily the right one.”[Paul]*

## Discussion

This study shows how NG doctors’ participation in a group coaching course contributes to their understanding of the organisational complexity of health care organisations. Participants realized the importance of building good relations, gradually began to see themselves as part of a team and acknowledged other staff members as valuable colleagues. The NGs learned to prevent or de-escalate conflicts by asking questions regarding the perspective of the other party rather than acting on own assumptions. Our research indicates that peer group coaching has particular advantages in addressing organisational issues, which are seldomly discussed in clinical departments or among doctors. The problems and perspectives presented by the participants and the subsequent thorough discussions provided a deeper understanding of the organisational complexity, and probably developed NGs’ capacity to handle organisational issues in a professional way.

### Socialisation – exploring the hidden and the medical culture

Professional discussions, morning conferences and handovers between shifts have been established working methods in the field of medicine for ages [28–30]. Doctors learn the “doctors’ language” and learn how to behave when listening to and discussing with peers [29]. In the morning conference, NGs experienced the “tone” of academic discussions, diagnostic reasoning and learned how to communicate in professional relations. This experience might give the NGs the impression that the medical culture is characterised by objectivity, value neutrality, technical expertise and authority as described by Apker et al. [29]. To gain access to the medical community you have to master the professional rhetoric. Not being able to master the cultural linguistic codes combined with inexperience can cause a NG doctor to perceive him/herself as standing outside the group.

Social identity theory describes how aspirants for membership of a group categorize and compare themselves to the fully-fledged members of the group during the process of striving for full membership [31, 32]. Our previous research. Our previous research [18] indicates that NGs are uncertain of their own status as doctors due to inexperience, uncertainty about own ability and a lack of affiliation. As known from social psychology, groups typically distinguish themselves through positive descriptions of the core values characterising the specific group (the “in-group”) while at the same time denoting the values of other groups (the “out-groups”) [31, 32]. Some NGs enter the medical profession with values that are quite different from the apparent values in some departments. This difference in values complicates the NG doctors’ move towards full membership in the “in-group”.

In the group coaching course, participants have the opportunity to discuss medical culture and ways of practising medicine in a detailed manner. In this respect the learning potential of these dialogues exceeds what might be observed at a morning conference in an academic setting. In the expression of hopes, dreams and expectations to professional life of a NG, the participants learned to see themselves as doctors (“in-group”) even though their values may be quite different from the stereotypes expressed during a morning report. The NGs realised that it is possible to be an “in-group” member while being true to important personal values at the same time [33]. This perspective made it easier to approach experienced colleagues and ask for necessary supervision and guidance.

### Relations and inter-professional communication

The importance of good and trusting relations became evident through listening to peers and sharing experiences. Participants became aware that good relations to senior doctors could help facilitate communication, allow them to feel appreciated and make it easier for them to ask for necessary feedback. Furthermore, these novice-expert relations could pave the way into the medical community. The formal introduction program offered to NGs in a department helped to reduce uncertainty. However, reducing uncertainty is not enough to ensure work engagement, which requires both positive social relations with colleagues and the experience of belonging to a group of professionals [34]. During the group coaching course, the NGs learned how to build positive and rewarding relations with colleagues in the department, which is instrumental to workplace learning, job satisfaction and organisational commitment [34–36].

The participants learned the importance of taking the perspective of other people into consideration. Some had experienced that asking for the perspective of others could dispel a looming crisis. Applying this skill in the collaboration with other professionals can potentially move the communication framework beyond that of a “tribal culture” [31, 32] and towards an interprofessional community. In the interprofessional culture, doctors are members of the team around the patient – a team where all perspectives are welcomed and discussed. Such teams may reduce the risk of mistakes and lead to a higher quality of health care [37].

### Emerging leadership

It is generally agreed that doctors need leadership training [7, 8, 38], but also that many doctors have too little or no leadership training [39]. The group coaching course provided the NGs with a more nuanced understanding of the organisational culture and helped them to navigate in the complex health care organisation. A positive side effect of attending the group coaching course was that some participants initiated small projects of organisational change as a result of the discussions and the insights gained at the course. Furthermore, leadership skills such as active listening, seeing the perspectives of others, conflict management, the ability to optimise collaboration and the courage to speak up and delegation of tasks were important learning outcomes of the course. In this way, the group-coaching course contributes to the development of leadership skills in NG doctors alongside other relevant roles or core competencies of a doctor [1–4].

### Strengths and limitations

The participants in this study were volunteers, which may introduce a bias when discussing the effects of the group-coaching course. Participants in the course might represent NGs who are curious and eager to learn or who feel a need to change their current coping strategies. The majority of participants were female, however, this proportion reflects the current intake in medical schools, where around 65% are now female.

In Denmark, we have a national culture of generally low power distance [40]. Despite this, a clear hierarchical structure and a perceived distance between junior and senior doctors as well as between doctors and other health care staff is still present in Danish health care organisations. Thus, we believe that the results of this study could be relevant in other countries despite differences in organisational and national cultures.

This study is based on self-reported changes in behaviour. However, the many examples of participants experimenting with new coping strategies in their own work place indicate that participation in the group coaching course made a positive difference in their daily working life.

We reached data saturation after analysing data from three of the six courses. However, including data from all six courses allowed for a broader diversity in participants and a richer data set from which to draw our conclusions.

## Conclusion

Newly graduated doctors seem to develop a growing awareness of organisational issues and a deeper understanding of the complexity of health care organisations through participation in a group coaching course. Participants report improvements in skills related to communication, building collegial relations, conflict management and leadership. All skills that might contribute to a more effective organisational practice among newly graduated doctors. Further studies are needed to consolidate the findings and explore possible effects in the organisation.

## Supplementary information

**Additional file 1.** Evaluation forms. Midterm and End of course evaluations forms. All questions included

**Additional file 2.** Letter of decision from Ethics committee. Letter from the Ethics committee, Central Denmark Region Committees on Health Research Ethics

## Data Availability

Due to fact the all participants have been guaranteed anonymity, unfortunately we cannot make the original interview transcripts available for others as this could endanger the anonymity of the participants. All data material is in Danish and a large part of data are notes on observations, where participants’ names are still readable. Raw data therefore cannot be shared. However, on reasonable request the tables from the first analysis could be provided, however, these are also in Danish.

## Abbreviation

NG: Newly graduated doctors
