# Supplementary material for: How group coaching contributes to organisational understanding among newly graduated doctors
Source: BMC Med Educ. 2020 Jun 16;20:193. doi: 10.1186/s12909-020-02102-8 (PMC7298786; doi:10.1186/s12909-020-02102-8)
Supplement: Supplementary file 1 — Additional file 1. Evaluation forms. Midterm and End of course evaluations forms. All questions included [file 12909_2020_2102_MOESM1_ESM.docx]

## Additional file 1

Questionnaire filled in by participants at midterm and at end of course.

| Midterm questionnaire |
| --- |
| What do you find important in participating in this group-coaching course? |
| What does the collegial community and the experiences reported by the other participants mean to you? |
| How does participation in this group-coaching course influence your daily work life (give specific examples)? |
| Do you think that other people (superiors, colleagues, patients, relatives) have noticed that you participate in the course? |
| What will be important (a focus) for you during the last part of the course? |

| End of course questionnaire |
| --- |
| What was your primary focus, and how will you describe your expectations to the group-coaching course in relation to the focus? |
| To what extent were your expectations fulfilled? (0 = not all; 10 = totally fulfilled) |
| What were the implications participating in the group coaching course regarding your focus? |
| How did participation in the group-coaching course influence your thoughts and actions in your interaction with patients and relatives? |
| How did participation in the group-coaching course influence your thoughts and actions in your interaction with colleagues in the department? |
| How did participation in the group-coaching course influence your thoughts and actions regarding your interaction with other collaborative partners? |
| How did participation in the group-coaching course influence your career planning? |
| What were the implications of participants coming from different departments? |
| How will you describe the importance of being in a group regarding your benefit from participating in the course? |
| Please rate the importance of the different elements in the course (0 = no importance; 10 = very important):  Group-coaching  Theoretical presentations  Reading theoretical articles  Informal networking |

*The answers to this specific question was used as data in the study
