# Supplementary material for: How group coaching contributes to organisational understanding among newly graduated doctors
Source: BMC Med Educ. 2020 Jun 16;20:193. doi: 10.1186/s12909-020-02102-8 (PMC7298786; doi:10.1186/s12909-020-02102-8)
Supplement: Supplementary file 2 — Additional file 2. Letter of decision from Ethics committee. Letter from the Ethics committee, Central Denmark Region Committees on Health Research Ethics [file 12909_2020_2102_MOESM2_ESM.pdf]

Regionshuset
Viborg

Regionsssekretariatet
Juridisk kontor
De Videnskabsetiske Komiteer
For Region Midtjylland

Skottenborg 26
DK-8800 Viborg
Tel. +45 7841 0183
komite@rm.dk
www.komite.rm.dk

Lydia de Lasson. overlæge, ph.d. Msci
Aarhus Universitetshospital
Operation og Intensiv Øst
Palle Juul-Jensens Boulevard 99
8200 Aarhus N

Dato 09-05-2016
Sagsbehandler Helle Nikkel
komite@rm.dk
Tel. +4578410186
Sagsnr. 1-10-72-6-16

Dear Lydia de Lasson

Thank you for your email in which you ask if your study "Professional identity formation in the transition from medical school to working life: A qualitative study of group-coaching courses for junior doctors" shall be notified to The Central Denmark Region Committees on Health Research Ethics.

According to the Act on Research Ethics Review of Health Research Projects, Act number 593 of 14 July 2011 section 14 (1) only health research studies shall be notified to the Committees. The Committees do not consider your study to be health research study (section 2 (1)) and therefore the study shall not be notified to the Committees.

Kind regards

Helle Nikkel

Helle Nikkel
Secretary

The Central Denmark Region Committees On Health Research Ethics
